# Analysis of 31-year-old patient with *SYNGAP1* gene defect points to importance of variants in broader splice regions and reveals developmental trajectory of *SYNGAP1*-associated phenotype: case report

**DOI:** 10.1186/s12881-017-0425-4

**Published:** 2017-06-02

**Authors:** Darina Prchalova, Marketa Havlovicova, Katalin Sterbova, Viktor Stranecky, Miroslava Hancarova, Zdenek Sedlacek

**Affiliations:** 1 Department of Biology and Medical Genetics, Charles University 2nd Faculty of Medicine and University Hospital Motol, Plzenska 130/221, 15000 Prague 5, Czech Republic; 2 Department of Child Neurology, Charles University 2nd Faculty of Medicine and University Hospital Motol, Prague, Czech Republic; 3 Institute of Inherited Metabolic Disorders, Charles University 1st Faculty of Medicine and General University Hospital, Prague, Czech Republic

**Keywords:** *SYNGAP1* gene, Intellectual disability, Epilepsy, Splice mutation, Splice region, Whole exome sequencing

## Abstract

**Background:**

Whole exome sequencing is a powerful tool for the analysis of genetically heterogeneous conditions. The prioritization of variants identified often focuses on nonsense, frameshift and canonical splice site mutations, and highly deleterious missense variants, although other defects can also play a role. The definition of the phenotype range and course of rare genetic conditions requires long-term clinical follow-up of patients.

**Case presentation:**

We report an adult female patient with severe intellectual disability, severe speech delay, epilepsy, autistic features, aggressiveness, sleep problems, broad-based clumsy gait and constipation. Whole exome sequencing identified a *de novo* mutation in the *SYNGAP1* gene. The variant was located in the broader splice donor region of intron 10 and replaced G by A at position +5 of the splice site. The variant was predicted *in silico* and shown experimentally to abolish the regular splice site and to activate a cryptic donor site within exon 10, causing frameshift and premature termination. The overall clinical picture of the patient corresponded well with the characteristic *SYNGAP1*-associated phenotype observed in previously reported patients. However, our patient was 31 years old which contrasted with most other published *SYNGAP1* cases who were much younger. Our patient had a significant growth delay and microcephaly. Both features normalised later, although the head circumference stayed only slightly above the lower limit of the norm. The patient had a delayed puberty. Her cognitive and language performance remained at the level of a one-year-old child even in adulthood and showed a slow decline. Myopathic facial features and facial dysmorphism became more pronounced with age. Although the gait of the patient was unsteady in childhood, more severe gait problems developed in her teens. While the seizures remained well-controlled, her aggressive behaviour worsened with age and required extensive medication.

**Conclusions:**

The finding in our patient underscores the notion that the interpretation of variants identified using whole exome sequencing should focus not only on variants in the canonical splice dinucleotides GT and AG, but also on broader splice regions. The long-term clinical follow-up of our patient contributes to the knowledge of the developmental trajectory in individuals with *SYNGAP1* gene defects.

**Electronic supplementary material:**

The online version of this article (doi:10.1186/s12881-017-0425-4) contains supplementary material, which is available to authorized users.

## Background

SYNGAP1 (synaptic Ras GTPase-activating protein 1) is localised in dendritic spines of neocortical pyramidal neurons. It is a component of the synaptic signalling regulation pathway. In this pathway, the N-methyl D-aspartate receptor (NMDAR) is activated by glutamate to allow the entry of Ca^2+^ ions into the postsynaptic space. This triggers calmodulin-dependent protein kinase II (CaMKII) phosphorylation. CaMKII then phosphorylates and activates SYNGAP1 which in turn activates Ras and Rap. This leads to endocytosis of the α-amino-3-hydroxy-5-methyl-4-isoxazolepropionic acid receptor (AMPAR), a major excitatory neurotransmitter receptor of the central nervous system, and repression of AMPAR trafficking to excitatory postsynaptic membrane [1]. The pathway is the key regulator of synaptic plasticity.

While mice with heterozygous knockout of the mouse *SYNGAP1* homologue show impaired behaviour and cognition [2], complete deficiency of the gene is lethal [3]. In humans, *de novo* heterozygous *SYNGAP1* mutations cause mild to severe intellectual disability (ID) [4, 5]. *SYNGAP1* mutation carriers often have generalised epilepsy, absent or severely delayed speech, aggressiveness, sleep problems and broad-based clumsy gait. About half of the patients show autistic behaviour. *SYNGAP1* appears to be one of the most frequently mutated ID-causing genes, with mutations possibly explaining 0.7 to 1% of ID [5]. Mutations were reported throughout the whole gene, with the exception of the most 5′ and 3′ exons. Until now, 45 different point mutations or indels have been described in 47 patients. Out of this, 21 mutations were frameshift, 13 were nonsense, 6 were splicing, 4 were missense and one was both missense and splicing [reviewed in 5]. The *SYNGAP1*-associated phenotype is most likely caused by haploinsufficiency of the *SYNGAP1* gene and its protein product [6].

In this report we describe an adult female patient with a *de novo* mutation in the *SYNGAP1* gene identified using whole exome sequencing (WES). The variant is in the splice donor region of intron 10 but it does not affect the canonical splice donor dinucleotide GT; instead it replaces G by A at position +5 of intron 10. The variant was predicted *in silico* and shown experimentally to affect splicing by abolishing the regular splice site and by activating a cryptic donor site within exon 10. Therefore this observation underscores the importance of considering variants not only in the canonical splice donor and acceptor dinucleotides GT and AG, but also in broader splice regions. While the overall phenotype of the patient is rather similar to the previously published patients with *SYNGAP1* defects, she is 31 years old which contrasts with most other published *SYNGAP1* cases, who were much younger. Thus our study contributes to the knowledge of development of the phenotype associated with *SYNGAP1* gene defects.

## Case presentation

### The subject

The currently 31-year-old female was first seen at our institution at the age of 16 years due to severe ID, epilepsy and autistic features. The previous history of the patient was reconstructed from medical records, photographs and parental interviews.

**Fig. 1:**
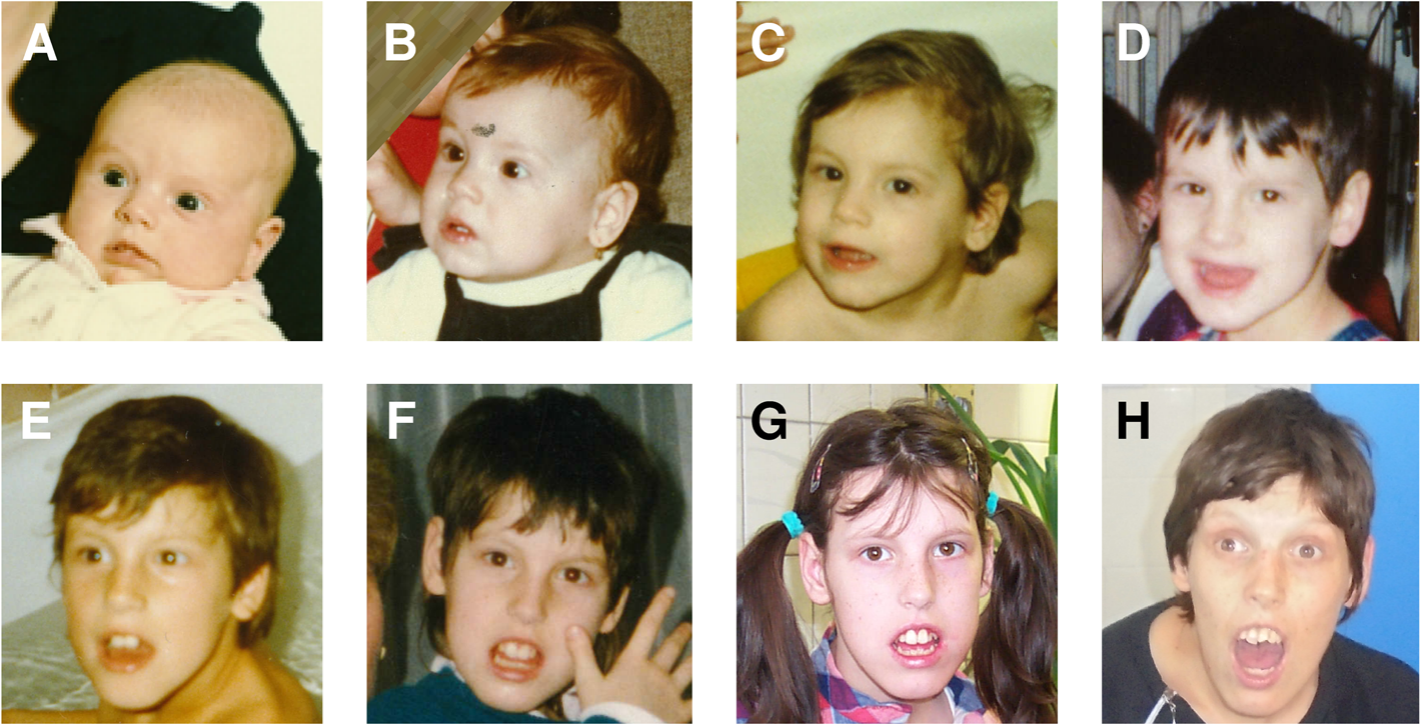
Facial photographs of the patient at the age of 1 month (**a**), 1 year (**b**), 3 years (**c**), 5 years (**d**), 7 years (**e**), 10 years (**f**), 16 years (**g**), and 31 years (**h**)

The patient (Fig. 1a-h) was born from an uneventful pregnancy of unrelated healthy parents (at birth of the girl the mother and father were aged 26 and 29 years, respectively). The patient had one older sister who suffered from anorexia nervosa. A paternal cousin of the mother of the patient had Down syndrome, the maternal grandmother of the mother of the patient suffered from multiple sclerosis, and a maternal aunt of the father of the patient had schizophrenia.

The development of the patient in the prenatal and perinatal periods was largely normal. The labour was spontaneous in the 42nd week of gestation. The birth weight was 3950 g (97th centile) and the length was 52 cm (75th centile). Postnatal adaptation was uncomplicated, except poor suck. The patient was breastfed shortly and throve on artificial nutrition till two years of age. At around three years of age a period of food refusal started, and the girl ended up on glucose infusions. Till eight years of age such periods repeated at least three times. Since eight years of age she has eaten almost everything, but she did not chew and her dependence on ground food worsened with age. Constipation has been a major problem since the age of one year, with unsuccessful anal sphincter surgery at the age of 14 years.

The developmental milestones of the girl have been significantly delayed from the age of six months despite physiotherapy. She stood up at the age of 12 months, and achieved independent sitting and walking without support at the age of 18 months and three years, respectively. Her gait was unsteady with frequent crashes. Currently her posture and gait resemble “crouch” gait with anteflexed neck and increased flexion of the knees, although this wide-based gait with bent knees was not observed till the age of 16 years. Her hands are dystonic and tremor appears with voluntary movement. The speech development started on time. The patient started crooning at the age of three months and pronouncing syllables at the age of 12 months. Later she started to use a few words, but her verbal skills have stopped at the level of a one-year-old child.

The patient had significant growth delay. The skeletal maturation and the onset of puberty were delayed by about four years. The first menstrual period occurred at the age of 18 years. In childhood and early adolescence the patient had a gracile habitus and microcephaly. At the age of 16 years her height was 155 cm (3rd centile), weight was 41 kg (below the 3rd centile), and head circumference (HC) was 51.5 cm (below the 3rd centile). At the age of 18 years the height was 162 cm (20th centile), weight was 50.7 kg (12th centile) and HC was not measured because of lack of cooperation. At the age of 28 years the height was 167 cm (48th centile), the weight was 58 kg (45th centile) and the HC was 53.1 cm (11th centile).

At the age of two months the patient developed strabism, which was corrected by surgery at the age of 13 months. The facial phenotype in early childhood was not remarkable with a possible exception of full cheeks and larger ears. Later she started to show open mouth appearance and a long narrow face. At the age of 16 years her facial dysmorphism included long hypomimic face, prominent chin, narrow almond-shaped palpebral fissures, long nose with prominent nasal bridge and overhanging columella, open mouth appearance, high arched palate, large upper teeth protruding from the mouth, wide lower lip and large ears. She had long cone-shaped fingers with 5th finger clinodactyly and haluces valgi. Since 28 years of age the myopathic facial features have become more prominent, the face has become longer and more coarse in appearance, and a protuberant chin has been more evident. Currently the patient has her mouth constantly open, with protruding upper incisors, and the teeth are irregularly stored. Dysarthria and hypersalivation are also present.

The patient was an irritable baby with back arching and frequent startle reactions. Seizures started at the age of two years. Ictal behavioural arrest with blinking was followed by head drop and fall. She never had any other type of seizures. Topiramate was the most effective drug in controlling the epileptic paroxysms although many other antiepileptic drugs have been used. Since being on topiramate medication, she has been completely free of seizures. EEG repeatedly showed generalized irregular SW discharges. Cerebral MRI at the age of 18 years was normal. She started to show self-harming behaviour at four years of age. She was hitting her head, and later around puberty she started biting herself which led to extensive bruising. Aggression became evident at preschool age and had a tendency to worsen with age. Since the age of 15 years the aggression has become uncontrollable, and the patient has been treated with a series of antidepressants and behaviour-damping medication. Currently aggressiveness appears only occasionally. As an infant she used to wake up several times at night and she continues to have problems with sleep initiation, and has a short sleep time.

At the age of five years a diagnosis of autism was first proposed, and later the girl was diagnosed with pervasive developmental disorder, not otherwise specified, with severe ID. Currently, at the age of 31 years, the patient suffers from severe to profound ID and hyperactivity. Autistic features were predominantly observed at the pre-school age. Although they improved with age and currently are less evident, the insistence on sameness persists as well as oversensitivity to certain sounds. On the other hand, the patient likes physical closeness and contact.

### Methods

Karyotyping was performed according to standard protocols. Genomic DNA of the patient was analysed using the Human CytoSNP-12 BeadChips (Illumina, San Diego, CA, USA) according to the manufacturer’s protocol. For WES of the family trio, SeqCap EZ Human Exome Library v3.0 (Roche NimbleGen, Madison, WI, USA) was used for exome capture and HiSeq1500 system (Illumina) was utilised for paired-end massive parallel sequencing (see Additional file 1 for details on microarray and WES data analysis).

The *SYNGAP1* variant and its *de novo* nature were confirmed by Sanger sequencing of fragments amplified from the DNA of the family trio using primers targeting exon 10 and intron 10. The bioinformatic prediction of the impact of the variant on splicing was performed using programs Human Splicing Finder, MaxEnt, NNSPLICE, NetGene2, ESEfinder, Alternative Splice Site Predictor (ASSP), Splice Port and Cryp-Skip (see Additional file 1 for details on primer sequences and data analysis). RNA was extracted from whole blood of the patient, her parents and normal controls using the PAXgene Blood RNA Kit (PreAnalytiX, Hombrechtikon, Switzerland). RT-PCR was performed using SuperScript III One-Step RT-PCR System with Platinum *Taq* DNA Polymerase (Invitrogen, Carlsbad, CA, USA) and primers targeting *SYNGAP1* exons 8–9 and 12. The resulting cDNA fragments were Sanger sequenced.

### Results

**Fig. 2:**
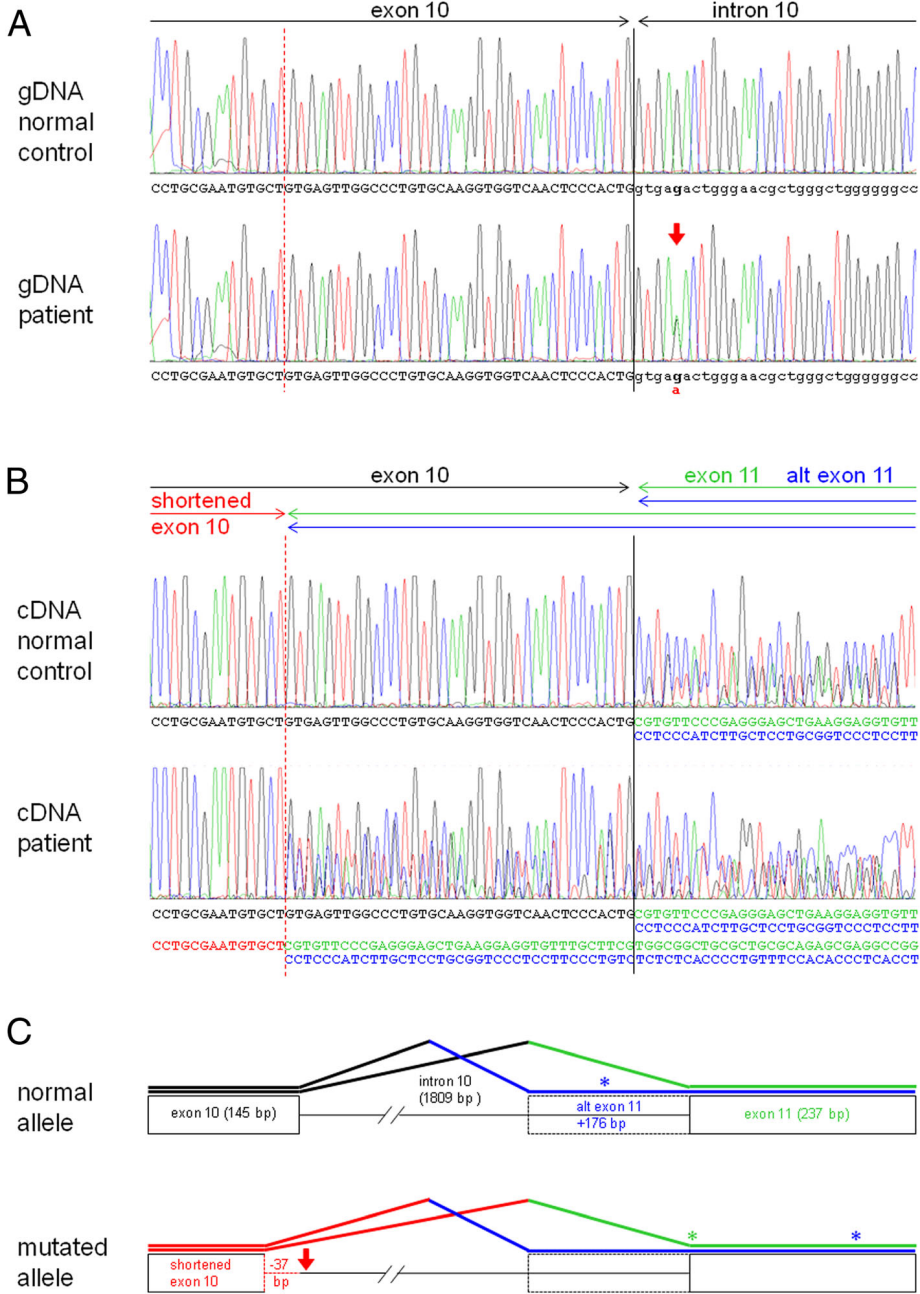
Electropherograms of Sanger sequences of *SYNGAP1* genomic DNA (gDNA) (**a**) and lymphocyte cDNA (**b**) of a representative normal control and the patient, and schematics of splicing of the exon 10 - exon 11 region in mRNA from the normal and mutated *SYNGAP1* alleles (**c**). The variant in intron 10 is marked by a *thick red arrow*. The regular exon 10 - intron 10 boundary and the new splice donor site in exon 10 are indicated by a *solid black vertical line* and a *dashed red vertical line*, respectively. *Black*, *red*, *green* and *blue* lettering in (**b**) and *thick lines* in (**c**) correspond to regular splicing of exon 10, aberrant splicing of exon 10, regular splicing of exon 11 and alternative splicing of exon 11, respectively. *Asterisks* indicate premature stop codons

Karyotyping and microarray analysis of the patient did not reveal any significant aberrations. WES of the family trio identified a single *de novo* heterozygous variant in the *SYNGAP1* gene (chr6:33406701, hg19, NM_006772.2:c.1676 + 5 G > A) in the patient, which was confirmed using Sanger sequencing (Fig. 2a). The variant was classified as a splice region variant as it affected position +5 of the splice donor of intron 10. The variant was absent in public variant databases. All tools used for the *in silico* analysis of the effect of the variant on splicing predicted disruption of the donor splice site of intron 10, and two tools which allowed also the prediction of the outcome (Cryp-Skip and ASSP) predicted the activation of a cryptic site in exon 10 (predicted to be 37 bp upstream of the regular site using ASSP) rather than skipping of the whole exon 10 (see Additional file 1 for details). Several much less strong candidate variants inherited by the patient from one of her unaffected parents were also identified and are listed in Additional file 1.

RT-PCR experiments with *SYNGAP1* cDNA primers detected novel splicing products in the patient which were absent in the parents and normal controls. The variant indeed activated the predicted cryptic splice donor site 37 bp upstream of the regular end of exon 10. Because of the existence of an alternative splice acceptor site in intron 10 located 176 bp upstream of the regular start of exon 11 (which was also predicted using ASSP), two transcripts could be detected in the parents and controls: the regular transcript with a regular junction of exons 10 and 11, and an alternative transcript where the end of exon 10 was joined to the last 176 bp of intron 10 (the alternative start of exon 11) (Fig. 2b-c). The alternative transcript contained a frameshift and a premature stop codon (p.Val560Leufs*34). In the patient, four different transcripts could be detected: the two described above and two which joined the activated cryptic donor site (thus lacking the last 37 bp of exon 10) with the regular and alternative start of exon 11. Both these transcripts also contained frameshifts and premature stop codons (p.Cys547Serfs*7 and p.Cys547Serfs*117).

## Discussion and conclusions

The *SYNGAP1* variant NM_006772.2:c.1676 + 5 G > A identified in our patient was the sole *de novo* variant found in her exome, it was absent in databases of normal genetic variation, and it was predicted and experimentally confirmed to have a disruptive effect on mRNA splicing. This together with the firmly established role of the *SYNGAP1* gene in ID and the phenotypic match between our patient and previously published patients with *SYNGAP1* defects strongly implicated the variant as causal for the disorder in our patient.

The variant affected an intronic position in the broader splice donor region. Contrary to variants in the well-known invariant splice site dinucleotides GT and AG, most variant prioritising pipelines do not assign the highest priority to variants in broader splice regions. The case of our patient clearly shows that these variants can also be detrimental for correct mRNA splicing. Particularly the intronic position +5 seems to be especially important, as the base G usually present in this position is among the most conserved in the consensus sequence of splice donor sites [7]. Based on the conservation patterns [7], at least variants in positions +3 to +6 and −3 to −20 should be explored in the broader splice donor and acceptor regions, respectively.

For the assessment of the effect of the variant on *SYNGAP1* mRNA splicing we used *in silico* prediction and laboratory analysis. Both approaches indicated a disruption of the regular splice donor site and activation of a cryptic site within exon 10, leading to frameshift and loss of function of the variant allele. An acceptor site 176 bp upstream of the regular start of exon 11 was also predicted, and the usage of this site was confirmed experimentally both in normal controls and in the patient. Database search indicated that the distal part of intron 10 was expressed also in multiple cell lines from the ENCODE project [8]. The inclusion of this segment results in premature termination, and the biological meaning of this alternative splicing event is unclear. Therefore we could observe a total of three transcripts with predicted premature termination codons, one in normal controls and three in the patient. As our experimental conditions did not block nonsense-mediated RNA decay (NMD) [9, 10], the transcripts seem not to be subject of NMD. This may reflect the fact that the efficiency of NMD in lymphocytes is lower than in other cell types [10]. It has to be stressed that our experiment using peripheral blood lymphocyte mRNA may not fully reflect the expression of *SYNGAP1* in the brain. Also, it is not clear if the transcripts with premature termination codons can indeed serve for the production of truncated proteins. Nevertheless, the variant *SYNGAP1* allele present in the patient definitely lost the capacity to produce the normal transcript and the full-length protein.

Our patient shared many clinical features with previously published patients [reviewed in 5]. While *de novo* mutations in *SYNGAP1* were initially reported to cause non-syndromic ID, later reports described more syndromic cases [4, 5, 11]. The phenotype of our patient supports the existence of a specific *SYNGAP1*-associated phenotype characterised predominantly by mild to severe ID, epilepsy, severe speech delay, aggressiveness, sleep problems, broad-based clumsy gait, autism and constipation. The development of our patient has also been delayed since infancy, and her motor and speech abilities stopped at the level of a one-year-old child. The seizures (head drops and eyelid myoclonia) were similar to other cases with *SYNGAP1* defects and no specific seizure triggers could be revealed. After initial polytherapy, long-term seizure freedom was achieved by topiramate. The behavioural disturbances observed such as restlessness, temper tantrums and aggression were noted also in other patients with *SYNGAP1* mutations, just as the sleep disorder (insomnia and frequent night-time awakenings). The gait of our patient was unsteady and she had intermittent tremor and dystonic posturing of her hands. Her face had myopathic features. Constipation has been a major issue in the everyday life of our patient since infancy. Although this feature was not reported among the characteristics in the latest patient series and review [5], earlier cases showed this symptom [11], and our patient supports the association of constipation with *SYNGAP1* defects. Genetic testing performed in our patient prior to WES included testing for Angelman syndrome which was also the case of patients reported previously [11]. This may indicate a possible phenotypic overlap and suggest that the *SYNGAP1*-associated phenotype could be considered a differential diagnosis to Angelman syndrome.

We could follow-up the patient till her adulthood and this allowed us to observe the developmental trajectory of her phenotype, contrary to most reports which described much younger patients. Between the age of one year and adulthood there was no sharp regression but rather a continuous slow decline in her cognitive and speech abilities. Autistic behaviour was most pronounced at preschool age and although later it showed some improvement, some symptoms are constantly present. While the seizures remained well-controlled, her aggressive behaviour worsened with age and required medication. Microcephaly which was observed in childhood gradually normalised and after puberty (which was significantly delayed) the HC has been slightly above the lower limit of the normal range. Other growth parameters have also normalised after puberty. With increasing age the facial appearance of our patient has become coarser and the myopathic facies with open mouth appearance and protruding teeth have become more pronounced. The gait problems worsened significantly in her teens. Thus our observations indicate that in our patient the developmental trajectory of the *SYNGAP1*-associated phenotype continues to show a specific dynamics in adolescence and adulthood.

## Additional file

Additional file 1: Supplementary methods, results and references. (PDF 283 kb)

## Abbreviations

AMPAR: α-amino-3-hydroxy-5-methyl-4-isoxazolepropionic acid receptor
ASSP: Alternative Splice Site Predictor
CaMKII: Calmodulin-dependent protein kinase II
HC: Head circumference
ID: Intellectual disability
NMD: Nonsense-mediated RNA decay
NMDAR: N-methyl D-aspartate receptor
WES: Whole exome sequencing
