# Supplementary material for: Analysis of 31-year-old patient with SYNGAP1 gene defect points to importance of variants in broader splice regions and reveals developmental trajectory of SYNGAP1-associated phenotype: case report
Source: BMC Med Genet. 2017 Jun 2;18:62. doi: 10.1186/s12881-017-0425-4 (PMC5457574; doi:10.1186/s12881-017-0425-4)
Supplement: Additional file 1: — Supplementary methods, results and references. (PDF 283 kb) [file 12881_2017_425_MOESM1_ESM.pdf]

SUPPLEMENTARY INFORMATION

Microarray and whole exome sequencing (WES) data analysis

The bioinformatic analysis of the microarray data was performed using Genome Studio (Illumina) [1] and QuantiSNP [2]. The WES reads were mapped to the reference genome using Novoalign [3]. SAM to BAM conversion and PCR duplicate removal were performed using Picard Tools [4]. Genome Analysis Toolkit (GATK) [5] was used for local realignment around indels, base recalibration, variant recalibration and genotyping. Variants were annotated using the GEMINI framework [6], and were filtered based on the population frequencies using several public databases (1000 Genomes Project [7], Exome Aggregation Consortium (ExAC) [8], Exome Sequencing Project [9] and Geuvadis European Exome Variant Server [10]) and an in-house database of population-specific variants. Filtering was also performed according to the predicted severity of the impact of the variants, inheritance patterns (*de novo*, X-linked (especially in boys), autosomal recessive (homozygous or compound heterozygous) or autosomal dominant (incomplete penetrance of inherited variants must be considered when parents are unaffected)), presence in in-house candidate gene lists and phenotype match with published mutation carriers. The most interesting candidate variants identified in the patient, which could potentially correlate with her phenotype, are listed in Supplementary Table 1. However, with the exception of the *SYNGAP1* variant they were inherited from unaffected parents. In addition, many were predicted to be tolerated, had higher frequencies in ExAC, or affected just one allele in autosomal recessive conditions. Different strategies were used for the detailed prediction of impact of missense and splice site/splice region variants. Prediction tools such as PolyPhen2 [11], SIFT [12], CADD score [13] and GERP [14] were used for missense variants. Splice site/splice region variants were analyzed using tools listed in Supplementary Table 2. OMIM (http://omim.org/) was used for analyses of gene - disease associations. The whole study used the hg19 coordinates.

Supplementary Table 1. List of interesting candidate variants identified in the patient.

gene	inheritance (OMIM)	OMIM	ClinVar	chr	position	ref	alt	impact	Polyphen2	SIFT	CADD	GERP	ExAC	inheritance
SYNGAP1	AD	Mental retardation, autosomal dominant 5	--	chr6	33406701	G	A	splice_region	--	--	12.96	4.84	--	*dn*
MBD5	AD	Mental retardation, autosomal dominant 1	other	chr2	149227826	A	C	missense	benign	tolerated	5.97	3.78	2/121322	mat
MTO1	AR	Combined oxidative phosphorylation deficiency 10	pathogenic	chr6	74191932	G	A	missense	probably damaging	damaging	35	5.48	28/121380	mat
RELN	AD	{Epilepsy, familial temporal lobe, 7}	uncertain	chr7	103138558	C	T	missense	benign	tolerated	19.09	5.89	1/121350	mat
CACNA1H	AD	{Epilepsy, idiopathic generalized, susceptibility to, 6}	--	chr16	1260783	G	A	splice_region	--	--	0.01	-8.48	372/115076	mat
ELP2	AR	Mental retardation, autosomal recessive 58	--	chr18	33721141	A	G	missense	benign	tolerated	12.67	1.61	--	mat
KDM5C	XLR	Mental retardation, X-linked, syndromic, Claes-Jensen type	--	chrX	53246446	C	T	missense	benign	tolerated	13.1	5.32	20/80084	mat
IGBP1	XLR	Corpus callosum, agenesis of, with mental retardation, ocular coloboma and micrognathia	--	chrX	69366608	T	C	missense	benign	tolerated	7.84	3.97	438/81943	mat
ARHGEF6	XLR	Mental retardation, X-linked 46	--	chrX	135763005	C	A	missense	probably damaging	damaging	18.05	4.9	--	mat
CACNA1E	AD	Not associated with a disorder in OMIM, candidate gene for intellectual disability and autism	--	chr1	181765954	G	A	missense	benign	deleterious	22	5.91	71/80968	pat

AD, autosomal dominant; AR, autosomal recessive; XLR, X-linked recessive; *dn*, de novo; mat, maternal; pat, paternal.
The ExAC column lists allele frequencies.

Supplementary Table 2 shows the prediction tools used in the analysis of splice site/splice region variants and results obtained using these tools for the *SYNGAP1* NM_006772.2:c.1676+5 G>A variant.

Supplementary Table 2. Prediction tools used and prediction results for the *SYNGAP1* variant.

**Tool**	**Suppl Reference**	**Scores**		**Comments**
		**WT**	**Variant**	
Human Splicing Finder	[15]	91.76	79.59	The score range is 0 to 100. The threshold score for a functional site is 65. If the score decreases by more than 10%, the variant is considered deleterious for the functioning of the splice site. The studied variant decreases the score by 13.26%.
MaxEnt	[16]	7.54	0.89	The score range is -20 to 20. The threshold score for a functional site is 3. If the score decreases by more than 30%, the variant is considered deleterious for the functioning of the splice site. The studied variant decreases the score by 88.2%.
NNSPLICE	[17]	0.93	-	The score range is 0 to 1. The studied variant is not recognised as a splice site and is assigned no score.
NetGene2	[18]	0.95	-	The score range is 0 to 1. The studied variant is not recognised as a splice site and is assigned no score.
ESEfinder	[19]	9.63	-	The threshold score for a functional site is 6.67. The studied variant is not recognised as a splice site and is assigned no score.
Alternative Splice Site Predictor (ASSP)	[20]	12.82	5.46	The score for the wild type site corresponds to a less than 5% probability of a false positive splice site. The score for the studied variant corresponds to a 20% probability of a false positive splice site.<br>The program also predicts the activation of a cryptic donor site 37 bp upstream of the regular site. The score of the cryptic site is 8.57, which also corresponds to a less than 5% probability of a false positive splice site.
SplicePort	[21]	0.85	-0.71	The score for the wild type site corresponds to a 0.19% probability of a false positive splice site. The score for the studied variant site corresponds to a 6.6% probability of a false positive splice site.
Cryp-Skip	[22]	n.a.	n.a.	Cryp-Skip predicts the outcome for an exon if it is affected by a splicing mutation. The Cryp-Skip score for exon 10 is 0.66 which means a higher probability of activation of a cryptic splice site compared to skipping of the whole exon 10.

WT, wild type; n.a., not applicable.

Analysis of the SYNGAP1 splice region variant

The analysis of the *SYNGAP1* NM_006772.2:c.1676+5 G>A splice region variant on genomic DNA and cDNA levels used primers listed in Supplementary Table 3.

Supplementary Table 3. Primers used in the study of the *SYNGAP1* variant.

**Primer location**	**Primer sequence**
Exon 10 primer used for DNA analysis	ATCCGTGCTCTGTATGAATC
Intron 10 primer used for DNA analysis	TATCTCAAAGCTCTGCCTTC
Exon 8-9 primer used for cDNA analysis	GTACAGGCAAGGCCAAGGACTTC
Exon 12 primer used for cDNA analysis	CAGAAACTCATTCATGAAGCCCAG

Supplementary References

1. GenomeStudio. Illumina, San Diego. http://www.illumina.com/techniques/microarrays/array-data-analysis-experimental-design/genomestudio.html. Accessed 20 May 2016.
2. Colella S, Yau C, Taylor JM, Mirza G, Butler H, Clouston P, et al. QuantiSNP: An objective bayes hidden-markov model to detect and accurately map copy number variation using SNP genotyping data. Nucleic Acids Res. 2007;35:2013–25.
3. Novoalign. Novocraft Technologies, Selangor. http://www.novocraft.com/products/novoalign. Accessed 11 July 2016.
4. Picard Tools. Broad Institute, Cambridge. http://broadinstitute.github.io/picard. Accessed 11 July 2016.
5. McKenna A, Hanna M, Banks E, Sivachenko A, Cibulskis K, Kernytsky A, et al. The Genome Analysis Toolkit: A MapReduce framework for analyzing next-generation DNA sequencing data. Genome Res. 2010;20:1297–303.
6. Paila U, Chapman BA, Kirchner R, Quinlan AR. GEMINI: Integrative Exploration of Genetic Variation and Genome Annotations. PLoS Comput. Biol. 2013;9.
7. Auton A, Abecasis GR, Altshuler DM, Durbin RM, Bentley DR, Chakravarti A, et al. A global reference for human genetic variation. Nature. 2015;526:68–74.
8. Lek M, Karczewski KJ, Samocha KE, Banks E, Fennell T, O AH, et al. Analysis of protein-coding genetic variation in 60,706 humans. 2016;536.
9. Exome Variant Server. NHLBI GO Exome Sequencing Project (ESP), Seattle. http://evs.gs.washington.edu/EVS/. Accessed 11 July 2016.
10. Geuvadis European Exome Variant Server. European Medical Sequencing Consortium, Barcelona. http://geevs.crg.eu/home. Accessed 11 July 2016.
11. Adzhubei IA, Schmidt S, Peshkin L, Ramensky VE, Gerasimova A, Bork P, et al. A method and server for predicting damaging missense mutations. Nat. Methods. 2010;7:248–9.
12. Ng PC, Henikoff S. Predicting Deleterious Amino Acid Substitutions. 2001;863–74.
13. Kircher M, Witten DM, Jain P, O'Roak BJ, Cooper GM, Shendure J. A general framework for estimating the relative pathogenicity of human genetic variants. Nat Genet. 2014;46:310–5.
14. Cooper GM, Stone EA, Asimenos G, Green ED, Batzoglou S, Sidow A. Distribution and intensity of constraint in mammalian genomic sequence. Genome Res. 2005;15:901–13.
15. Desmet F-O, Hamroun D, Lalande M, Collod-Béroud G, Claustres M, Béroud C. Human Splicing Finder: an online bioinformatics tool to predict splicing signals. Nucleic Acids Res. 2009;37:e67.
16. Yeo G, Burge CB. Maximum Entropy Modeling of Short Sequence Motifs with Applications to RNA Splicing Signals. J. Comput. Biol. 2004;11:377–94.
17. Reese MG, Eeckman FII, Kulp D, Haussler D. Improved Splice Site Detection in Genie. J. Comput. Biol. 1997;4:311–23.
18. Brunak S, Engelbrecht J, Knudsen S. Prediction of human mRNA donor and acceptor sites from the DNA sequence. J Mol Biol. 1991;220:49–65.
19. Cartegni L, Wang J, Zhu Z, Zhang MQ, Krainer AR. ESEfinder: A web resource to identify exonic splicing enhancers. Nucleic Acids Res. 2003;31:3568–71.
20. Wang M, Marín A. Characterization and prediction of alternative splice sites. Gene. 2006;366:219–27.
21. Dogan RI, Getoor L, Wilbur WJ, Mount SM. SplicePort-An interactive splice-site analysis tool. Nucleic Acids Res. 2007;35:1–7.
22. Divina P, Kvitkovicova A, Buratti E, Vorechovsky I. Ab initio prediction of mutation-induced cryptic splice-site activation and exon skipping. Eur. J. Hum. Genet. 2009;17:759–65.
